# Case report: A complicated course of Collet-Sicard syndrome after internal carotid artery dissection and lenticulo-striatal artery infarction

**DOI:** 10.3389/fneur.2022.939236

**Published:** 2022-10-20

**Authors:** Dennis A. Nowak, Rainer Linden, Peggy Arnold, Veronika Seitz, Katrin Stangl, Christina Wendl, Felix Schlachetzki

**Affiliations:** ^1^Vamed Klinik Kipfenberg, Kipfenberg, Germany; ^2^Neurologische Klinik und Poliklinik, Universitätsklinikum Marburg, Marburg, Germany; ^3^Klinik für Neurologie, Medbo Bezirksklinikum Regensburg, Universität Regensburg, Regensburg, Germany; ^4^Institut für Neuroradiologie, Medbo Bezirksklinikum Regensburg, Universität Regensburg, Regensburg, Germany

**Keywords:** carotid artery dissection, dysphagia, dysphonia, skull base pathology, dysphagia and rehabilitation

## Abstract

A 40-year-old Caucasian man presented with sudden onset of left-sided hemiparesis associated with dysphonia, dysphagia, and right-sided weakness on shoulder elevation and head rotation. The clinical examination revealed deviation of the tongue to the right, absence of right-sided gag reflex, right-sided palatal and vocal cord paresis, and weakness of the right trapezius and sternocleidomastoid muscles; all were in addition to left-sided brachiocephalic-accentuated hemiparesis. The diagnostic examination revealed dissection of the right carotid artery with occlusion of the middle cerebral artery and infarction in the lenticular-striatal artery territory. Mechanical thrombectomy with stent angioplasty of the right internal carotid artery was performed. The paresis of the left side of the body completely regressed within a week after symptom onset, but the dysphonia, weakness of the right trapezius and sternocleidomastoid muscles, and especially dysphagia persisted and regressed slowly but gradually. The patient required percutaneous gastric tube feeding for the next 12 weeks, possibly because of involvement of subcortical white matter tracts. The constellation of symptoms and clinical findings were consistent with Collet-Sicard syndrome, an extremely rare disorder caused by direct compression of the caudal cranial nerves at the base of the skull.

## Introduction

Collet-Sicard syndrome is a very rare clinical condition characterized by combined paralysis of cranial nerves IX through XII (1, 2). The syndrome is usually caused by mass lesions at the base of the skull involving the jugular foramen and hypoglossal canal (1, 2). Usually, sympathetic fibers are spared. Neoplasms or traumas are the most common cause of Collard-Sicard syndrome (3), whereas dissection of the internal carotid artery is a very rare cause (4).

## Case report

A 40-year-old left-handed Caucasian man was admitted with sudden onset of moderate left hemiparesis, hoarseness, dysphagia, and weakness on lifting the right shoulder and turning the head to the left. There was no evidence of Horner syndrome. The medical history was unremarkable. He was not taking any regular medications. He reported no major or minor head or neck trauma and no recent infection.

**Figure 1:**
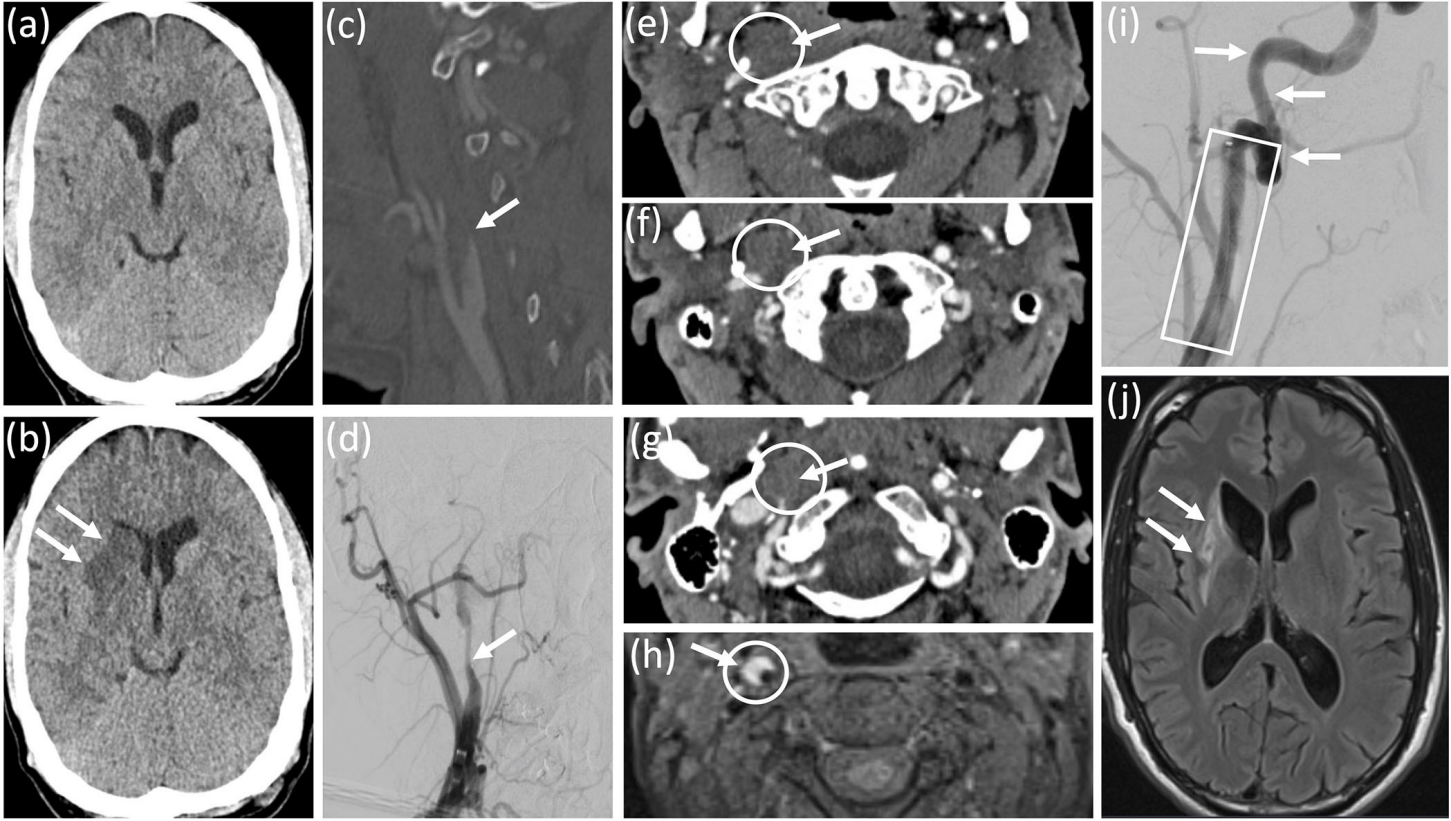
Diagnostic work-up and follow-up. **(a)** Initial computed tomography of the brain was unremarkable. **(b)** Computed tomography of the brain 24 h after symptom onset revealed an incomplete ischemic lesion within the territory of the right lenticulo-striatal arteries. Computed tomography angiography **(c)** and conventional cerebral angiography **(d)** revealed a filiform stenosis of the right internal carotid artery about 1.5 cm from the carotid bifurcation. **(e–g)** transversal computed tomography scans showed an intramural haematoma of the right internal carotid artery. **(h)** Transversal T1-weighted magnetic resonance imaging with fat suppression confirmed mural haematoma of the right internal carotid artery. **(i)** Conventional angiography after intra-arterial mechanical thrombectomy and stent implantation illustrated no residual stenosis of the right internal carotid artery. **(j)** Fluid attenuated inversion recovery magnetic resonance imaging 10 weeks from symptom onset revealed the ischemic lesion within the territory of the right lenticulo-striatal arteries, but no additional infarction.

The clinical examination revealed left-sided hemiparesis, most pronounced in the arm and face, right-sided tongue weakness, right-sided palatal and vocal cord paresis, right-sided palatal hyposensitivity, and paresis of the right trapezius and sternocleidomastoid muscles. Aphasia was not present. The computed tomography of the brain 3 h after symptom onset was unremarkable (Figure 1a). Additional computed tomography-angiography of the cerebral vessels revealed an incomplete, filiform occlusion of the right internal carotid artery ~1.5 cm distal to the carotid bifurcation (Figure 1c) and thrombus in the middle cerebral artery. These findings were confirmed by conventional angiography (Figure 1d). Transverse computed tomography scans showed wall swelling of the right internal carotid artery due to extensive intramural hematoma (Figures 1e–g). The transverse T1-weighted magnetic resonance imaging with fat suppression 10 weeks after symptom onset confirmed the mural hematoma of the right internal carotid artery (Figure 1h). Intravenous thrombolysis, followed by stent implantation within the right internal carotid artery, followed by mechanical thrombectomy was performed (Figure 1i). The detailed anamnestic re-evaluation revealed no causative evidence of carotid artery dissection. One day after the onset of symptoms, the computed tomography of the brain showed an incomplete ischemic lesion in the right lenticulostriatal arteries (Figure 1b), which was confirmed by magnetic resonance imaging with attenuated inversion recovery 10 weeks after the onset of symptoms (Figure 1j).

Fiberoptic examination of swallowing function was performed on admission. Penetration and aspiration of liquids and all foods were noted. Within 7 days of symptom onset, the left-sided hemiparesis regressed, leaving only mild deficits in the fine motor function of the hand. The dysphagia, dysphonia, hyposensitivity of the palate, and moderate weakness of the right trapezius and sternocleidomastoid muscles persisted but gradually regressed. Ten weeks after the onset of symptoms, the patient was still fed *via* a percutaneous gastric tube. Twelve weeks after the onset of symptoms, the swallowing function was restored and the percutaneous feeding tube was removed. Limited tongue mobility and mild hoarseness with nasal speech were still present.

## Discussion

Lesions of the cranial nerves occur in up to 12% of extracranial dissections of the internal carotid artery (4). Collet-Sicard syndrome is a rare clinical condition characterized by combined palsy of the lower cranial nerves, namely, the glossopharyngeus, vagus, accessorius, and hypoglossus nerves, in the absence of ipsilesional miosis, ptosis, or enophthalmus as sympathetic nerval structures are spared (1, 2). The patho-anatomical mechanism most likely to result in combined lower cranial nerve palsy after internal carotid artery dissection is direct compression due to an intramural haematoma at the level of the jugular foramen, where all nerves run through the upper carotid artery sheath (4, 5). Sympathetic nerve fibers also travel through the upper carotid artery sheath at this level, and in case combined lower cranial nerve palsy is associated with signs of Horner's syndrome, the clinical condition should be named Villaret syndrome (6). Therapeutic strategies for Collet-Sicard syndrome should focus on treatment of the underlying pathology as it primarily determines outcome (4, 5).

The prognosis of lower cranial nerve impairment in Collet-Sicard syndrome is generally considered good to excellent (5) but depends on the underlying pathology and additional lesions responsible for dysphagia. In our case, the impact of acute stent implantation on the submandibular portion of the internal carotid artery required for mechanical recanalization remains unclear (7, 8). In patients with stroke and dysphagia, it is generally debated whether one hemisphere is more dominant than the other within the complex bilateral neural network responsible for swallowing. Also, in addition, several lesion sites can potentially cause dysphagia, including the insula, basal ganglia, somatosensory and motor cortices, and internal capsule (9, 10). In our case, infarction of the right basal ganglia occurred in a left-handed person. It may be speculative, but the central lesion may have been responsible for the prolonged recovery of the dysphagia aspect of Collet-Sicard syndrome.

Additional neurophysiologic studies such as electromyographic examination of the muscles innervated by the inferior cranial nerves (trapezius, tongue muscles, and laryngeal muscles) or functional MRI were not performed in our case but could have provided additional information about the nature and severity of the peripheral nerve fiber damage and the brain lesion.

## Data availability statement

The raw data supporting the conclusions of this article will be made available by the authors, without undue reservation.

## Ethics statement

Written informed consent was obtained from the individual for the publication of any potentially identifiable images or data included in this article.

## Author contributions

DN and FS drafted the manuscript. RL, PA, VS, KS, and CW added clinical data and revised the manuscript. All authors contributed to the article and approved the submitted version.

## Conflict of interest

The authors declare that the research was conducted in the absence of any commercial or financial relationships that could be construed as a potential conflict of interest.

## Publisher's note

All claims expressed in this article are solely those of the authors and do not necessarily represent those of their affiliated organizations, or those of the publisher, the editors and the reviewers. Any product that may be evaluated in this article, or claim that may be made by its manufacturer, is not guaranteed or endorsed by the publisher.
